# Researching the molecular mechanisms of Taohong Siwu Decoction in the treatment of varicocele-associated male infertility using network pharmacology and molecular docking: A review

**DOI:** 10.1097/MD.0000000000034476

**Published:** 2023-08-04

**Authors:** Bo Wu, Xiaohong Lan, Xuqing Chen, Qinyan Wu, Yang Yang, Yuekun Wang

**Affiliations:** a Department of Pharmacy, Jinling Hospital, Medical School of Nanjing University, Nanjing, China.

**Keywords:** mechanism, molecular docking, network pharmacology, Taohong Siwu Decoction, varicocele-associated male infertility

## Abstract

Taohong Siwu Decoction (THSWD) was widely used for the treatment of varicocele-associated male infertility. However, the pharmacological mechanism of action is not completely clear. Therefore, network pharmacology and molecular docking were performed to explore potential mechanism of THSWD in the treatment of varicocele-associated male infertility. The Traditional Chinese Medicine Systems Pharmacology (TCMSP), Swiss Target Prediction, and GeneCards were used to retrieve candidate compounds, action targets, and disease-related targets. The construction of the protein–protein interaction (PPI) network and the screening of core genes were completed by the STRING and Cytoscape 3.9.1, respectively. The DAVID was used to obtain results of gene ontology function and Kyoto Encyclopedia of Genes and Genomes (KEGG) pathway enrichment analysis. The Mcule analysis platform was used to perform molecular docking. There were a total of 53 candidate compounds and 782 relevant targets in THSWD. There were 45 common targets between THSWD, varicocele, and male infertility, and 23 core genes were found in the PPI network. Biological processes involved response to hypoxia, regulation of blood pressure, cellular response to hypoxia, and regulation of the nitric oxide biosynthetic process. Furthermore, the KEGG pathway enrichment analysis showed that the common targets mainly regulated the disease of varicocele-associated male infertility through the HIF-1 signaling pathway, PI3K-Akt signaling pathway, Relaxin signaling pathway, and TNF signaling pathway. Finally, the molecular docking showed that luteolin, quercetin, and kaempferol had good intercalation with major targets. As predicted by network pharmacology, THSWD regulated varicocele-associated male infertility through multiple compounds and targets, and its mechanism was closely related to inflammatory response, reactive oxygen species damage, and function of blood vessels.

## 1. Introduction

Male infertility accounts for 60% of total infertility cases, and the common pathological causes of male infertility are varicocele and chronic reproductive system infections.^[1]^ Varicocele has been reported to be attributable to 35% to 40% of cases of male infertility.^[2]^ Surgery and medication are often used at this stage of clinical treatment, with medication being the main method. Taohong Siwu Decoction (THSWD) is composed of 6 Chinese herbs (Persicae Semen, CarthamiFlos, Rehmanniae Radix Praeparata, Paeoniae Radix Alba, Chuanxiong Rhizoma, and Angelicae Sinensis Radix), which have the activity of removing blood stasis, clearing collaterals, and nourishing blood. It is often used in patients with varicocele, and can effectively relieve the symptoms of testicular swelling, perineal swelling and pain, and the bulging of testicular tendons. Nevertheless, the pathogenesis of THSWD treatment for varicocele-associated male infertility is not fully understood. Therefore, exploring the mechanism of action of THSWD in the treatment of varicocele-associated male infertility was beneficial to clinically rational drug use and the development of new drugs.

**Figure 1.**
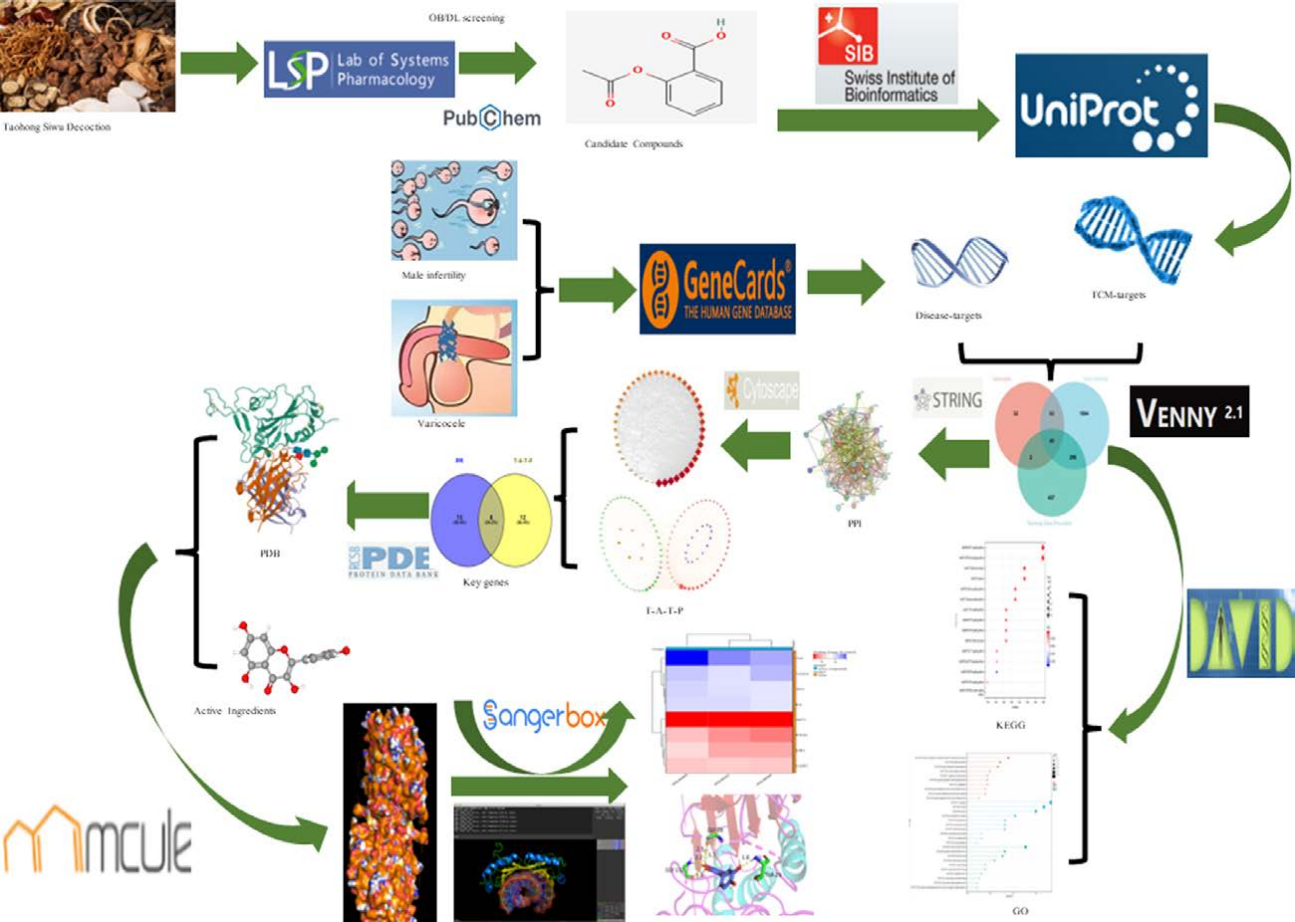
Flow chart of the pharmacological study of Taohong Siwu Decoction in the treatment of varicocele-associated male infertility.

In recent years, network pharmacology has been widely used to elucidate the mechanism of action of traditional Chinese medicine (TCM) in the treatment of diseases, and to screen the active ingredients and key targets of TCM by constructing a network of compounds and targets.^[3,4]^ As we all know, molecular docking technology plays a key role in drug design, development, and research. By calculating the binding energy of different conformations (small molecules binding to protein receptors), it is convenient to explore potential active compounds for the treatment of diseases.^[5]^ Therefore, we chose network pharmacology and molecular docking methods to study the mechanism of action of THSWD on varicocele-associated male infertility, excavate the potential active ingredients and key targets, and provide a theoretical basis for further research. The detailed technical flow of the study is shown in Figure 1.

## 2. Materials and methods

### 2.1. Candidate compounds and their associated targets were screened from THSWD

The chemical compounds of Persicae Semen (PS), Carthami Flos (CF), Rehmanniae Radix Praeparata (RRP), Paeoniae Radix Alba (PRA), Chuanxiong Rhizoma (CR) and Angelicae Sinensis Radix (ASR) were obtained from the Traditional Chinese Medicine Systems Pharmacology (TCMSP) (https://old.tcmsp-e.com/tcmsp.php), respectively. The candidate compounds and related targets were screened according to the screening conditions (oral bioavailability > 30, drug like > 0.18). Compounds in the TCMSP database have no relevant targets, and Swiss Target Prediction (http://swisstargetprediction.ch/) was used for target prediction. The SMILES of the compound were retrieved in the PubChem database (https://pubchem.ncbi.nlm.nih.gov/), and then the SMILES were entered into the search column in the Swiss Target Prediction database (http://www.swisstargetprediction.ch/), the target information was predicted, and the target with a probability greater than 0 was selected as the compound-related target. Finally, the predicted targets were corrected with the UniProt database (https://www.uniprot.org/).

### 2.2. Acquisition of targets about varicocele, male infertility, and common targets

The GeneCards database was used to obtain disease-related targets. Key words such as “varicocele” and “male infertility” were input into the search column to obtain target information. Subsequently, the targets were ranked by score, and the scores above the median were selected as the targets of varicocele and male infertility. The targets of THSWD, varicocele, and male infertility were uploaded to the online analysis tool of Venny2.1 to screen out the common targets of the three.

### 2.3. Construction of the protein–protein interaction (PPI) network and selection of core genes

The STRING database (https://cn.string-db.org/) was used for functional enrichment analysis of protein-protein interactions. The above common targets were uploaded to the STRING database, and “multiple proteins” was selected as the analysis module, “homo sapiens” as the species, and 0.4 as the minimum required interaction score. Other parameters were kept as defaults and searched. The obtained results were imported into Cytosape 3.9.1 software for visualization, and its built-in “Network Analyzer” tool module was used for topological network analysis.

### 2.4. Enrichment analysis of gene ontology (GO) and Kyoto Encyclopedia of Genes and Genomes (KEGG)

The DAVID database (https://david.ncifcrf.gov/tools.jsp) is widely used for annotation of biological information, which helps researchers discover biological internal connections. The common targets were uploaded to “gene list” in DAVIA database, and “gene symbol” was selected as an identifier. The screening false discovery rate of less than 0.05 was used as the result of GO and KEGG enrichment. The GO-related results were mainly composed of biological process (BP), cell components (CC), and molecular function (MF).

### 2.5. Construction of traditional Chinese medicine-active compounds-targets-pathway (T-A-T-P) network

In order to study the multi-target and multi-pathway therapeutic characteristics of TDSWD active compounds in the treatment of varicocele-associated male infertility, we constructed a “T-A-T-P” relationship network map using Cytoscape 3.9.1 software for biological network visualization and data integration analysis. The degree of a node in the network reflects the role that the node plays in the network, and if the degree is higher, the node plays the main bridge role in the network.

### 2.6. Molecular docking of active compounds to key targets

The RCSB database (https://www.rcsb.org/) stores a large amount of 3D structural data of biological macromolecules for research in basic biology, health, energy, education, and biotechnology. To obtain the PDB ID of target proteins, we inputted target proteins into the RCSB database. Mculle (https://mcule.com/) is an online drug discovery platform for drug research. 1-Click Docking (https://mcule.com/search/) is a functional module of Mculle for predicting ligand-target affinity. We only need to input the SMILES of the ligand and the PDBID of the receptor into the database to obtain 5 docking morphologies, and select the morphology with the lowest binding energy as the optimal docking. Finally, we visualized the molecular docking results using the PyMOL 2.5 (https://pymol.org/).

## 3. Results

### 3.1. Screening for the candidate compounds of THSWD by the database and analysis platform of TCMSP

A total of 53 candidate compounds that met the screening conditions in THSWD were selected, including 21 in PS, 20 in CF, 9 in PRA, 7 in CR, 2 in ASR, and 2 in RRP. beta-sitosterol was found in PS, CF, PRA, and ASR; kaempferol was found in CF and PRA; stigmasterol was found in CF, RRP, and ASR; and 3-epi-beta-sitosterol was found in RRP, PRA, and CR. Candidate compounds were presented in Table 1.

**Table 1:** Candidate compound information for Taohong Siwu Decoction.

No.	MOL_ID	Compound name	ADME		TCM
			OB (%)	DL	
1	MOL001323	alpha1-sitosterol	43.28	0.78	PS
2	MOL001329	(1R,2R,4S,5S,8S,9S,10R,11R)-4,5-dihydroxy-11-methyl-6-methylidene-16-oxo-15-oxapentacyclo[9.3.2.15,8.01,10.02,8]heptadec-12-ene-9-carboxylic acid	88.08	0.53	PS
3	MOL001339	gibberellin A119	76.36	0.49	PS
4	MOL001340	gibberellin A120	84.85	0.45	PS
5	MOL001342	(1R,2S,3S,4R,7S,10R,12S,13R)-12-hydroxy-4-methyl-14-methylidene-5-oxo-6-oxapentacyclo[11.2.1.14,7.01,10.03,9]heptadec-8-ene-2-carboxylic acid	72.70	0.54	PS
6	MOL001343	1-Methyl-4aalpha,6beta-Dihydroxy-8-Methylenegibba-3-Ene-1alpha,10beta-Dicarboxylic Acid 1,4a-Lactone	64.79	0.50	PS
7	MOL001344	(1R,2S,3S,4R,7S,10R,12R,13R)-12-hydroxy-4-methyl-14-methylidene-5-oxo-6-oxapentacyclo[11.2.1.14,7.01,10.03,9]heptadec-8-ene-2-carboxylic acid	88.11	0.54	PS
8	MOL001348	gibberellin A17	94.64	0.49	PS
9	MOL001349	gibberellin A19	88.60	0.46	PS
10	MOL001350	gibberellin A30	61.72	0.54	PS
11	MOL001351	gibberellin A44	101.61	0.54	PS
12	MOL001352	gibberellin A54	64.21	0.53	PS
13	MOL001353	gibberellin A60	93.17	0.53	PS
14	MOL001355	gibberellin A63	65.54	0.54	PS
15	MOL001358	gibberellin A7	73.80	0.50	PS
16	MOL001360	gibberellin A77	87.89	0.53	PS
17	MOL001361	gibberellin A87	68.85	0.57	PS
18	MOL001368	3-O-p-coumaroylquinic acid	37.63	0.29	PS
19	MOL000296	hederagenin	36.91	0.75	PS
20	MOL000358	beta-sitosterol	36.91	0.75	PS/CF/PRA/ASR
21	MOL000493	campesterol	37.58	0.71	PS
22	MOL001771	clionasterol	36.91	0.75	CF
23	MOL002680	flavoxanthin	60.41	0.56	CF
24	MOL002694	kinobeon A	48.47	0.36	CF
25	MOL002695	ethyl (1S,2R,3S)-6,7-dimethoxy-3-methyl-4-oxo-1-(3,4,5-trimethoxyphenyl)-2,3-dihydro-1H-naphthalene-2-carboxylate	43.32	0.65	CF
26	MOL002698	[(1R,3aR,5aR,5bR,7aR,9R,11aR,11bR,13aR,13bR)-3a,5a,5b,8,8,11a-hexamethyl-1-prop-1-en-2-yl-1,2,3,4,5,6,7,7a,9,10,11,11b,12,13,13a,13b-hexadecahydrocyclopenta[a]chrysen-9-yl] hexadecanoate	33.98	0.32	CF
27	MOL002710	pyrethrin II	48.36	0.35	CF
28	MOL002712	6-hydroxykaempferol	62.13	0.27	CF
29	MOL002714	baicalein	33.52	0.21	CF
30	MOL002717	3,5-Dihydroxy-2-[(2E)-3-(4-Hydroxyphenyl)Prop-2-Enoyl]Cyclohexa-2,5-Diene-1,4-Dione; 3,5-Dihydroxy-4-[(E)-3-(4-Hydroxyphenyl)Prop-2-Enoyl]Cyclohexa-3,5-Diene-1,2-Dione; 3,5-Dihydroxy-2-[(2E)-3-(4-Hydroxyphenyl)-1-Oxo-2-Propen-1-Yl]-2,5-Cyclohexadiene-1,4-Dione	51.03	0.20	CF
31	MOL002719	carthamidin	33.23	0.24	CF
32	MOL002721	quercetagetin	45.01	0.31	CF
33	MOL002757	7,8-dimethyl-1H-pyrimido[5,6-g]quinoxaline-2,4-dione	45.75	0.19	CF
34	MOL002773	beta-carotene	37.18	0.58	CF
35	MOL002776	baicalin	40.12	0.75	CF
36	MOL000422	kaempferol	41.88	0.24	CF/PRA
37	MOL000449	stigmasterol	43.83	0.76	CF/RRP/ASR
38	MOL000006	luteolin	36.16	0.25	CF
39	MOL000953	cholesterol	37.87	0.68	CF
40	MOL000098	quercetin	46.43	0.28	CF
41	MOL000359	3-epi-beta-sitosterol	36.91	0.75	RR/PRA/CR
42	MOL001919	palbinone	43.56	0.53	PRA
43	MOL001921	lactiflorin	49.12	0.80	PRA
44	MOL001924	paeoniflorin	53.87	0.79	PRA
45	MOL001925	paeoniflorgenin	68.18	0.40	PRA
46	MOL000211	betulinic acid	55.38	0.78	PRA
47	MOL000492	cianidanol	54.83	0.24	PRA
48	MOL001494	mandenol	42.00	0.19	CR
49	MOL002135	myricanone	40.60	0.51	CR
50	MOL002140	perlolyrine	65.95	0.27	CR
51	MOL002151	senkyunone	47.66	0.24	CR
52	MOL002157	wallichilide	42.31	0.71	CR
53	MOL000433	folic acid	68.96	0.71	CR

ASR = Angelicae Sinensis Radix, CF = Carthami Flos, CR = Chuanxiong Rhizoma, DL = drug like, OB = oral bioavailability, PRA = Paeoniae Radix Alba, PS = Persicae Semen, RRP = Rehmanniae Radix Praeparata, TCM = traditional Chinese medicine.

### 3.2. Common targets for acquisition of THSWD and varicocele-associated male infertility

**Figure 2.**
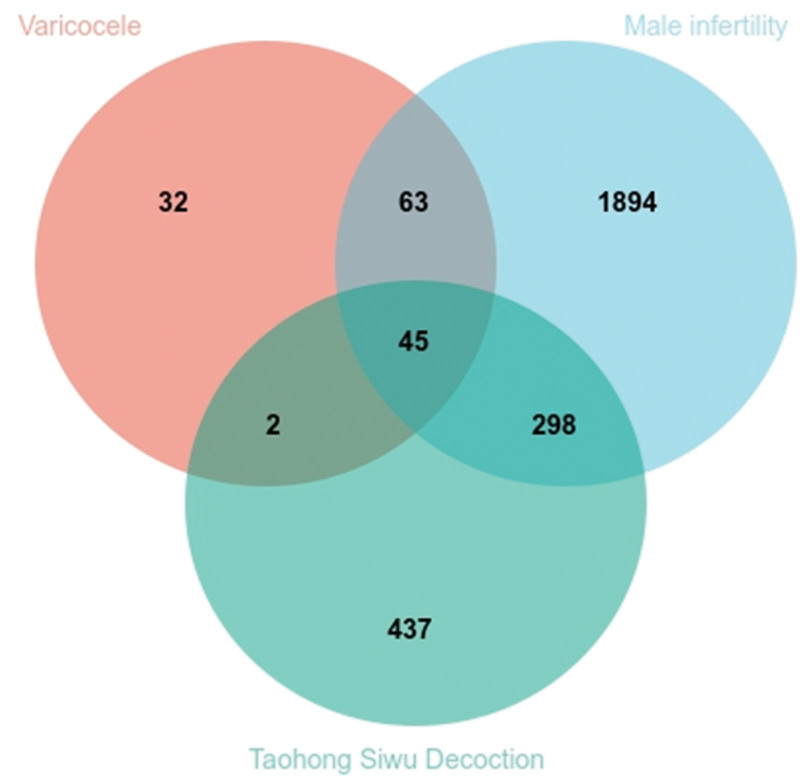
Common targets Venn diagram of Taohong Siwu Decoction, varicocele, and male infertility.

The predicted targets of 53 candidate compounds in THSWD were 782. In the database of GeneCards, 143 and 2301 targets were screened for varicocele and male infertility, respectively. A Venn diagram shows that there are 45 common targets among target predictions of candidate compounds in THSWD, varicocele, and male infertility (Fig. 2).

### 3.3. Construction and visualization analysis of the PPI network

**Figure 3.**
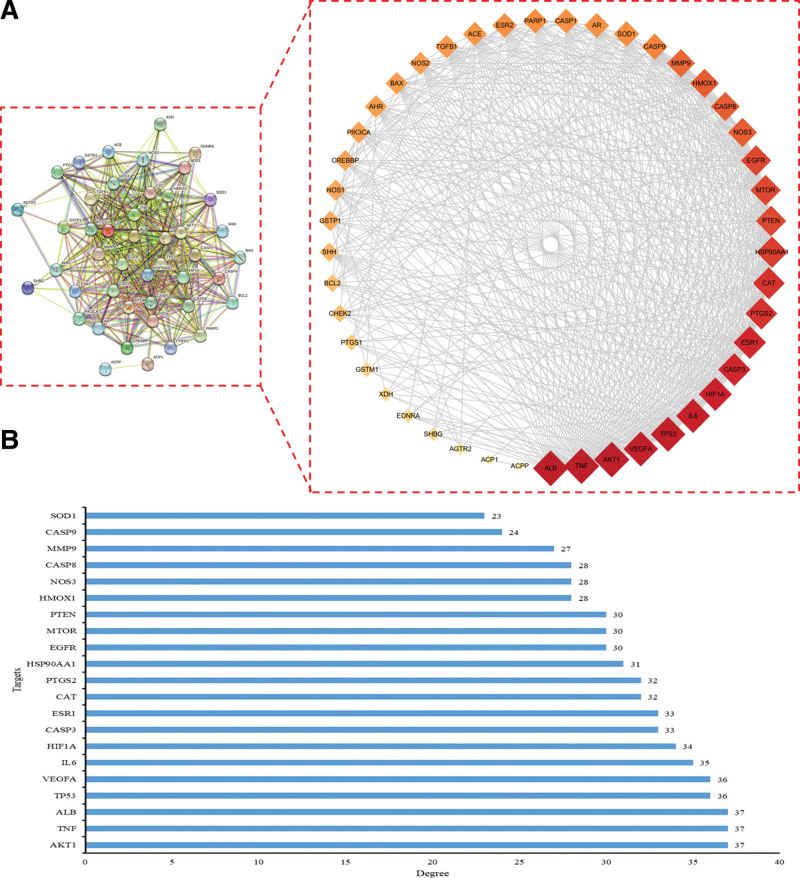
Cytoscape 3.9.1 software was used to screen the core targets in the PPI network. (A) The visualization of the PPI network was processed by Cytoscape 3.9.1 software. The diamond represents the target, and the size and color of the shape become progressively larger and darker with increasing degrees. (B) Degree of the core targets in the PPI network. PPI = protein–protein interaction.

To explore the interactions between proteins, a PPI network was constructed by the STRING database (Fig. 3A). There were 45 nodes and 499 edges in the network, and the average degree value was 22.2. In order to find the core genes of network, Cytoscape 3.9.1 software was used for PPI network diagram visualization. The node whose degree were greater than the average value were AKT1 (37), TNF (37), ALB (37), TP53 (36), VEGFA (36), IL6 (35), HIF1A (34),CASP3 (33), ESR1 (33), CAT (32), PTGS2 (32), HSP90AA1 (31), EGFR (30), MTOR (30), PTEN (30), HMOX1 (28), NOS3 (28), CASP8 (28), MMP9 (27), CASP9 (24), and SOD1 (23) (Fig. 3B), which may play an important role in the treatment of THSWD with varicocele-associated male infertility.

### 3.4. GO function and KEGG pathway enrichment analysis of common targets

**Figure 4.**
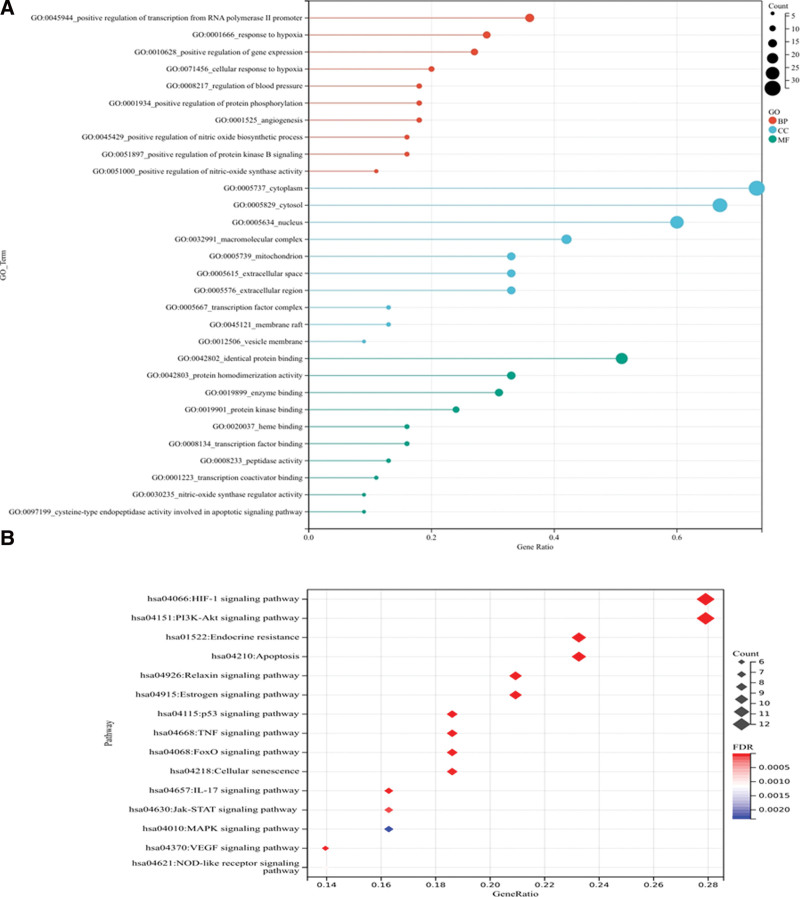
GO and KEGG enrichment analysis of common targets. (A) Lollipop plot of common target GO analysis. (B) KEGG pathway enrichment analysis of common targets. BP = biological process, CC = cell components, GO = gene ontology, KEGG = Kyoto Encyclopedia of Genes and Genomes, MF = molecular function.

GO functional enrichment included BP, CC, and MF. The GO functions of common targets were enriched in 183 BP, 21 CC and 43 MF (*P* < .05). The higher the gene ratio of an item, the more likely it is that the item played a major role in the GO function. Therefore, the top 10 items of the BP, CC, and MM gene ratio were selected to make a stick chart (Fig. 4A). As shown in the figure, BP mainly involves response to hypoxia, regulation of blood pressure, positive regulation of gene expression, angiogenesis, cellular response to hypoxia, positive regulation of the nitric oxide biosynthetic process, etc. CC mainly involves macromolecular complexes, cytoplasm, cytosol, mitochondrion, nucleus, etc. MF mainly involves enzyme binding, identical protein binding, protein homodimerization activity, protein kinase binding, heme binding, etc. In addition, a total of 139 items were enriched by KEGG analysis (*P* < .05), mainly involved pathways related to HIF-1 signaling pathway, PI3K-Akt signaling pathway, endocrine resistance, apoptosis, Relaxin signaling pathway, etc (Fig. 4B).

### 3.5. T-A-T-P network was constructed to analyze key genes

**Figure 5.**
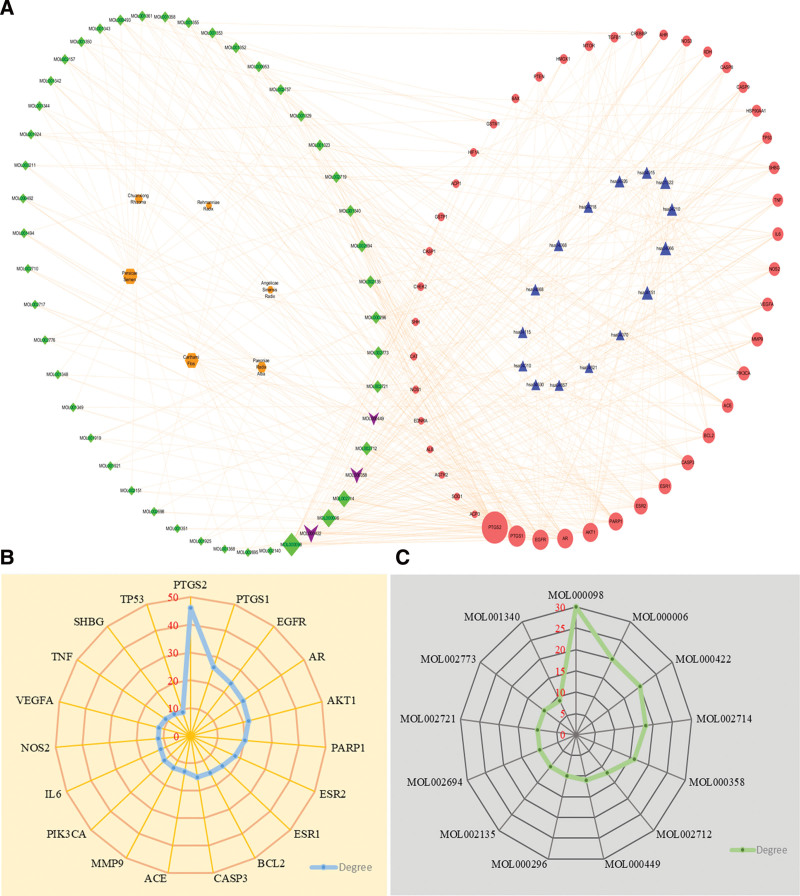
Cytoscape 3.9.1 software was used to analyze the information on targets and active ingredients in the T-A-T-P network. (A) Green diamond represents the active ingredient, a brown diamond represents TCM, a purple inverted triangle represents the same compounds in TCM, a red circle represents a common target, blue triangle represents a related pathway, and the magnitude increases with the degree value. (B and C) Degrees of targets and active compounds in the T-A-T-P network, respectively. TCM = traditional Chinese medicine, T-A-T-P = traditional Chinese medicine-active compounds-targets-pathway.

Information about nodes on the network was analyzed by the Network Analyzer plug-in in Cytoscape 3.9.1 software. As shown in Figure 5A, nodes represent medicinal materials, active ingredients, targets, or related pathways, while edges represent the relationships among medicinal materials, ingredients, targets, and pathways. The T-A-T-P network contained 113 nodes and 491 edges and the average degree was 8.7. The node degrees of the active compounds that were greater than the average degree were quercetin (MOL000098), kaempferol (MOL000422), luteolin (MOL000006), baicalein (MOL002714), beta-sitosterol (MOL000358), 6-hydroxykaempferol (MOL002712), stigmasterol (MOL000449), hederagenin (MOL000296), Kinobeon A (MOL002694), quercetagetin (MOL002721), beta-carotene (MOL002773), myricanone (MOL002135), and gibberellin A120 (MOL001340) (Fig. 5C). The composition degree values of quercetin, kaempferol, and luteolin were relatively high, indicating that they have a relatively important position in the network and may be the main active ingredient of THSWD in the treatment of varicocele-associated male infertility. The degree of 20 targets were above average, which were PTGS2 (46), PTGS1 (26), EGFR (24), AR (23), AKT1 (22), PARP1 (20), ESR2 (18), ESR1 (16), CASP3 (15), BCL2 (15), ACE (13), PIK3CA (13), MMP9 (13), NOS2 (12), IL6 (12), VEGFA (12), TNF (11), SHBG (10), TP53 (9) and HSP90AA1 (9) (Fig. 5B). Combined with the results of PPI network analysis, PTGS2, EGFR, AKT1, ESR1, CASP3, MMP9, IL6, VEGFA, TNF, TP53, and HSP90AA1 may be the key targets of THSWD in the treatment of varicocele-associated male infertility.

### 3.6. Molecular docking prediction of active ingredients and key targets in the treatment of varicocele-associated with male infertility by THSWD

**Figure 6.**
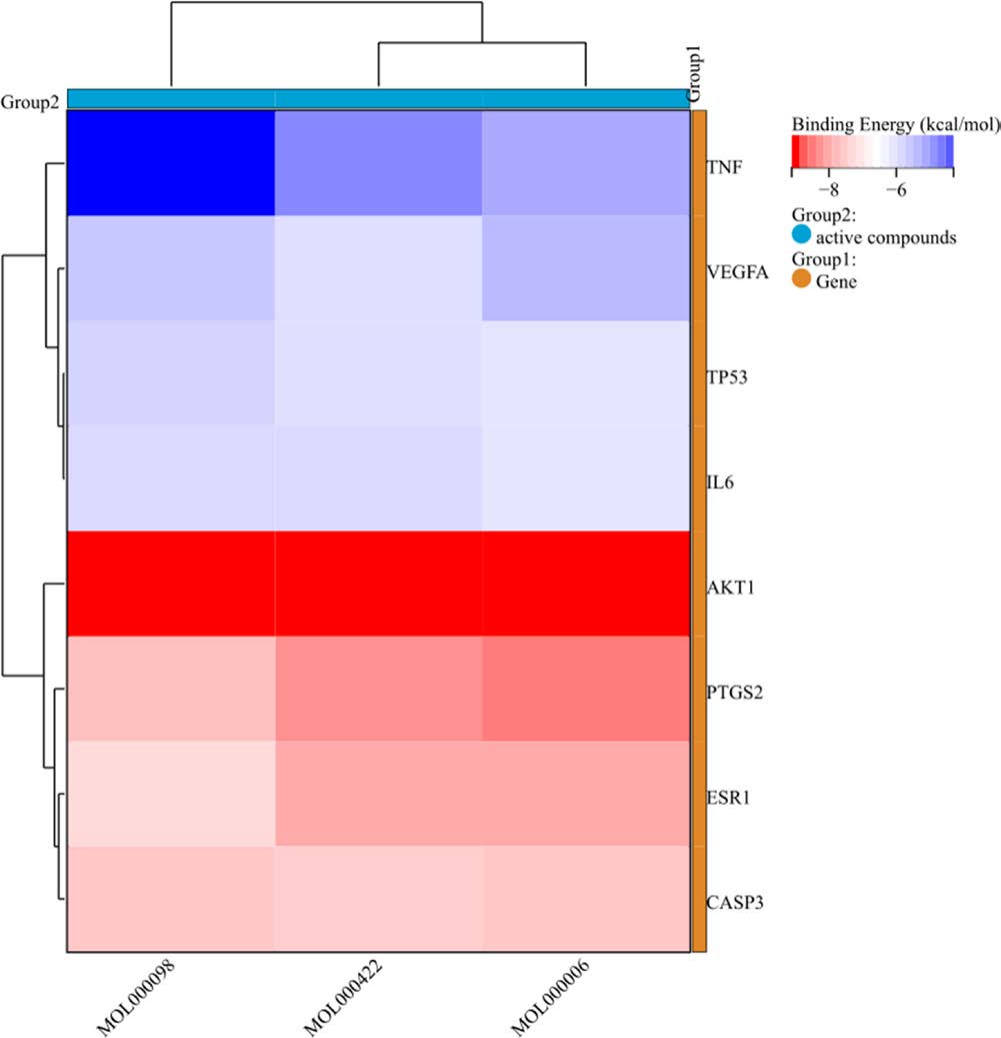
Heat map of molecular docking between active ingredients and key targets.

**Figure 7.**
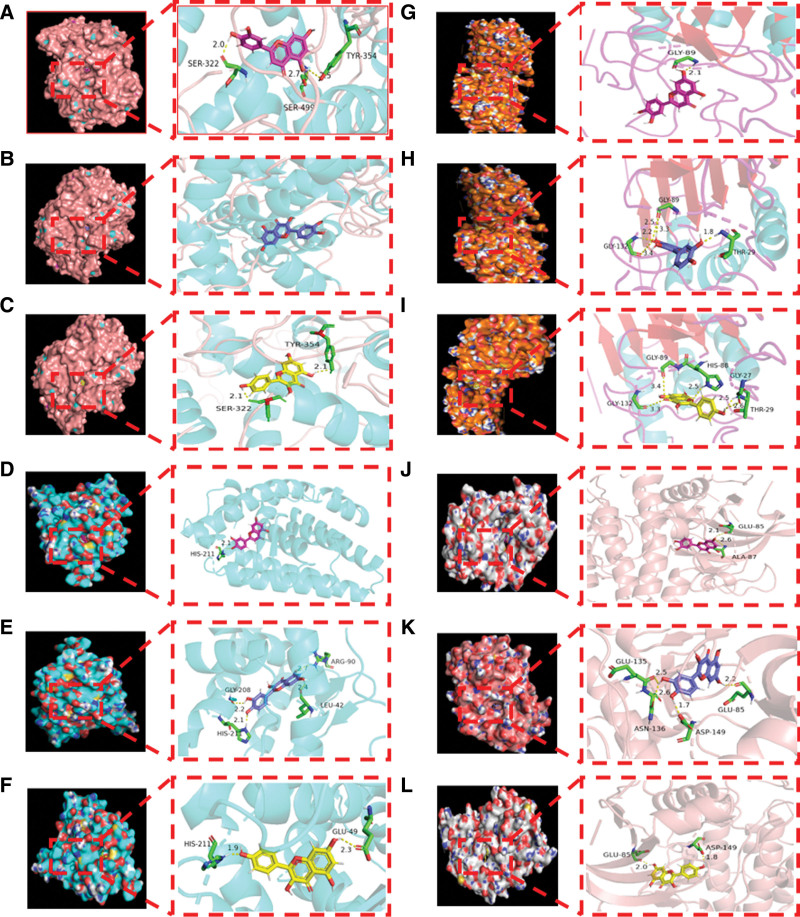
Optimal binding map for molecular docking between active ingredients of Taohong Siwu Decoction and key genes. (A, D, G, and J) Molecular docking diagrams of luteolin with PTGS2, ESR1, CASP3, and AKT1, respectively. (B, E, H, and K) Molecular docking diagrams of quercetin with PTGS2, ESR1, CASP3, and AKT1, respectively. (C, F, I, and L) Molecular docking diagrams of kaempferol with PTGS2, ESR1, CASP3, and AKT1, respectively.

The top 3 active ingredients in the T-A-T-P network were selected for molecular docking verification with 8 key targets (such as PTGS2, AKT1, ESR1, CASP3, IL6, VEGFA, TNF, and TP53) that were closely related. Previous studies have reported that the closeness of protein binding to ligand correlates with the binding energy, with binding energy below −5.0 kcal/mol indicating good binding and binding energy below −7.0 kcal/mol indicating very firm binding. Docking results showed that quercetin, kaempferol and luteolin were tightly bound to target proteins (binding energy < −5 kcal/mol), and binding energies to PTGS2, AKT1, ESR1, and CASP3 were lower than those of IL6, VEGFA, TNF, and TP53 (Fig. 6, Table 2). In order to more intuitively reflect the binding of the active ingredients to the key target, the results were visualized using the PyMOL 2.5, which can observe the amino acid residues and hydrogen bonds binding to the active compound. Therefore, active ingredients that bind strongly to target proteins were selected for visualization (Fig. 7). Luteolin binds to PTGS2 (amino acid residues of SER-322, SER-499, and TYR-354), ESR1 (amino acid residues of HIS-211), CASP3 (amino acid residues of GLY-89), and AKT1 (amino acid residues of GLU-85 and ALA-87), respectively (Fig. 7A, D, G, and J). Quercetin binds to PTGS2 (no residues), ESR1 (amino acid residues of HIS-211, GLY-208, LEU-42, and ARG-90), CASP3 (amino acid residues of GLY-132, GLY-89, and THR-29), and AKT1 (amino acid residues of GLU-135, ASN-136, ASP-149, and GLU-85), respectively (Fig. 7B, E, H, and K). Kaempferol binds to PTGS2 (amino acid residues of SER-322, and TYR-354), ESR1 (amino acid residues of HIS-211 and GLU-49), CASP3 (amino acid residues of GLY-132, GLY-89, HIS-88, GLY-27, and THR-29) and AKT1 (amino acid residues of GLU-85 and ASP-149), respectively (Fig. 7C, F, I, and L).

**Table 2:** The results of molecular docking between active ingredients of Taohong Siwu Decoction and key target proteins.

Active compounds	Affinity (kcal/mol)							
	PTGS2 (4RRW)	AKT1 (3CQU)	ESR1 (1L2I)	CASP3 (3DEI)	IL6 (1P9M)	VEGFA (1MJV)	TNF (5UUI)	TP53 (3LH0)
MOL000098	−7.8	−9.1	−7.4	−7.7	−6.0	−5.7	−4.3	−5.9
MOL000422	−8.4	−9.1	−8.1	−7.6	−6.0	−6.1	−4.9	−6.1
MOL000006	−8.6	−9.1	−8.1	−7.7	−6.2	−5.5	−5.3	−6.2

MOL000098 = quercetin, MOL000422 = kaempferol, MOL000006 = luteolin.

## 4. Discussion

Varicocele is not rare in clinical male diseases, patients are often accompanied by scrotal swelling discomfort, congestion swelling and pain symptoms, and even testicular atrophy and abnormal semen, which lead to male infertility and seriously affect the health of patients.^[6]^ Previous studies have shown that TCM for promoting blood circulation and removing blood stasis has a certain effect on varicocele.^[7]^ Other studies have shown that THSWD has analgesic, blood circulation, anti-inflammatory, anti-oxidative damage and other effects.^[8–10]^ Therefore, we speculate that THSWD may achieve its purpose of treating varicocele-associated male infertility by improving the blood circulation of the spermatic vein and increasing the blood supply to the testis and scrotum.

In this study, a total of 53 candidate compounds from THSWD were found to meet the screening criteria (oral bioavailability was 30% or greater and drug like was 0.18 or greater) from TCMSP. Among the candidate compounds, PS, CF, and PRA accounted for 39.6%, 37.7%, and 17%, respectively. PS maintained homeostasis through bidirectional regulation of plasma cAMP concentration and PKA protein in rats with hot and cold blood stasis.^[11]^ CF can improve blood circulation and is often used in cardiovascular and cerebrovascular diseases. In addition, it has been reported that CF can promote the production of NO in RAW 264.7 cells and reduce the proportion of neutrophils in the lung of mice.^[12,13]^ PRA plays a powerful anti-inflammatory role by regulating the body’s immune process, reducing the development of rheumatoid arthritis, liver fibrosis, and asthma.^[14–17]^ Combining previous studies, we speculate that PS, CF, and PRA play major roles in THSWD.

782 targets related to the candidate components were obtained from the DrugBank and Swiss Target Prediction databases. Subsequently, the target genes related to varicocele and male infertility in the GeneCards database were intersected with the genes related to candidate compounds, and 45 common targets were obtained. The PPI network composed of 45 common targets showed that 21 targets had higher than average degrees, and the highest ranking targets were AKT1, TNF, ALB, TP53, VEGFA, and IL6, respectively. AKT serine/threonine kinase 1 is a serine/threonine protein kinase, a member of the AGC kinases family, which participates in a variety of biological processes, including cell proliferation, metabolism, survival, signal transduction, and angiogenesis. It has been found that Akt can promote cell survival by affecting the activities of caspase-9, YAP, Bcl-2, and Bcl-x. It also increases VEGF secretion and mediates eNOS phosphorylation, vasodilation, and angiogenesis.^[18]^ Tumor necrosis factor (TNF) is an important pro-inflammatory factor in the body, that plays an important role in the inflammatory response and is often used as a therapeutic target for rheumatoid arthritis, inflammatory bowel disease, and a variety of cancers.^[19]^ Serum albumin encodes the most abundant protein in human blood, which not only regulates plasma colloid osmotic pressure, but also serves as a carrier protein for a variety of endogenous molecules, including hormones, fatty acids, metabolites, and exogenous drugs.^[20]^ Vascular endothelial growth factor A (VEGFA) is a member of the platelet-derived growth factor/VEGF family, which can mediate increased vascular permeability, induce angiogenesis and endothelial cell growth, promote cell migration, and inhibit cell apoptosis. Zou et al^[21]^ found that emodin promoted the proliferation of seryl-trna synthetase, reduced VEGFA expression, and inhibited tumor angiogenesis in triple-negative breast cancer. As a marker of inflammation, interleukin 6 (IL6) is involved in a series of inflammatory responses in the body. Inhibition of its expression can effectively reduce the inflammatory response and reduce the damage caused by inflammation to the body.^[22]^ Statement of defense: PPI network analysis showed that the main functions of the common targets were the regulation of cell proliferation, apoptosis, inflammation, vasodilation, and angiogenesis. To further verify the regulatory roles of these common targets, GO biological process and KEGG pathway enrichment analyses were performed.

Basically consistent with the above studies, BP was mainly involved in response to hypoxia, regulation of blood pressure, cellular response to hypoxia, and regulation of the nitric oxide biosynthetic process. KEGG pathway enrichment analysis showed that THSWD treatment of varicocele-associated male infertility disease was mainly through HIF-1 signaling pathway, PI3K-Akt signaling pathway, Relaxin signaling pathway, endocrine resistance, apoptosis, Estrogen signaling pathway, and TNF signaling pathway, etc. Previous studies^[23]^ have shown that A plays a regulatory role under hypoxic conditions in tissues and organs by reducing oxygen consumption or increasing the release of NO or other vasodilators. The relaxin signaling pathway is involved in the regulation of vascular and smooth muscle contraction. In contrast, the PI3K-Akt signaling pathway and the TNF signaling pathway are involved in the regulation of the inflammatory response, which have been widely studied in cancer in recent years.^[24,25]^

To figure out which active ingredients were involved in regulating the disease of varicocele-associated male infertility, we constructed the network of T-A-T-P. By analyzing the network, it was found that the degree of quercetin, kaempferol, luteolin, and baicalein in the network was higher, suggesting that these compounds play an important bridge role in the regulatory network, and indicating that this class of compounds plays a major role in the treatment of diseases. Quercetin is present in various fruits, vegetables, and Traditional Chinese Medicine. A large number of studies have shown that it has anti-oxidative, anti-inflammatory, anti-proliferative, anti-cancer, and anti-diabetic properties. In recent years, many researchers have found that it can lower blood pressure.^[26]^ Serban et al^[27]^ evaluated the effect of quercetin on blood pressure by systematic review and meta-analysis of randomized controlled trials, and found that quercetin had significant statistical significance in reducing blood pressure, and a daily dose of more than 500 mg was required. From the same source as quercetin, kaempferol is found in various vegetables, fruits, and plants and has anti-inflammatory properties. By regulating vascular endothelial inflammation, kaempferol has therapeutic effects on colitis, chronic hepatitis, lung injury, cancer, and diabetes.^[28]^ Feng et al^[29]^ found that different concentrations of kaempferol (5, 10 or 20 μM) induced G protein-coupled estrogen receptor activation, increased cell viability to nearly 10%, 19.8%, and 30%, respectively, and reduced the generation of cellular reactive oxygen species (16.7%, 25.6%, and 31.1%), thereby alleviating postmenopausal atherosclerosis. Yao et al^[30]^ found that kaempferol had the ability to protect the vascular endothelium in a mouse model of vascular injury by reducing oxidative stress mediated by H_2_O_2_ and paraquat (in vivo and in vitro). In addition, kaempferol inhibited the levels of TNF-α and IL-6 and the activation of NF-κB in aortic tissues and human umbilical vein endothelial cells. Luteolin is a kind of natural brass, and numerous studies have shown that it has anti-inflammatory, antioxidant, and anti-cancer biological activities.^[31–33]^ Boeing et al^[34]^ found that luteolin could reduce the oxidative stress response caused by iritantan by reducing reactive oxygen species and enhancing endogenous antioxidants. It also alleviated inflammation by reducing TNF, IL-1β and IL-6 levels and increasing IL-4 and IL-10 levels. In addition, it was found that Luteolin could also reduce the LPS-induced inflammatory injury of cardiomyocytes and the excessive release of inflammatory cytokines.^[35,36]^ Baicalein, a flavonoid originally extracted from the TCM scutellaria baicalensis, has a variety of pharmacological effects, such as inhibiting the oxidative stress response, inhibiting the cell apoptosis, inhibiting the inflammatory response, protecting mitochondria, and enhancing the expression of neuronal protective factors.^[37]^ Liu et al^[38]^ found that baicalein inhibited isoproterenol-induced cardiac hypertrophy in mice, and in vitro cell experiments found that Baicalin increased the expression of catalase and mitochondrial autophagy receptor FUN14 domain 1, promoted autophagy, cleared active oxygen species, and reduced isoproterenol-induced myocardial hypertrophy. Song et al^[39]^ found that baicalin could improve the cell viability of SH-SY5Y cells treated with MPP^+^ and reduce the accumulation of iron and lipid peroxides induced by MPTP, and the mechanism was closely related to inhibition of oxidative stress. In summary, the above studies showed that the main pharmacological effects of active ingredients were anti-oxidative stress, anti-inflammation, and improvement of blood circulation. In summary, the above studies showed that the main pharmacological effects of the active ingredients were anti-oxidative stress, anti-inflammation, and improvement of blood circulation, which was basically the same as our previous hypothesis.

Subsequently, to find out how closely the active ingredients bind to the key targets, we selected the top 3 compounds for molecular docking experiments. The results showed that luteolin, quercetin and kaempferol bind closely to PTGS2, ESR1, CASP3 and AKT1, respectively. These results indicate that these 3 compounds can be used as the main active ingredients of THSWD in the treatment of varicocele-associated male infertility.

## 5. Conclusion

In this study, a total of 53 candidate compounds of THSWD were obtained. The active ingredients of THSWD including quercetin, kaempferol, luteolin, baicalein, and beta-sitosterol, and PTGS2, EGFR, AKT1, ESR1, CASP3, MMP9, IL6, VEGFA, TNF, TP53, and HSP90AA1 were predicted as major targets for the treatment of varicocele-associated male infertility. Molecular docking results showed that luteolin, quercetin and kaempferol had good binding effects on major targets. KEGG pathway enrichment analysis showed that THSWD may play a therapeutic role through HIF-1, PI3K-Akt, and Relaxin signaling pathways. In summary, our study further improves the mechanism of action of THSWD in the treatment of varicocele-associated male infertility, and provides scientific evidence for subsequent experimental research and clinical rational administration.

## Acknowledgments

We thank Director Ming Gao for her valuable comments and support for this study. Xiwei Song, Xingdong Wang, and Min Huang are not among the authors, but I would like to thank them for their assistance with data processing.

## Author contributions

**Data curation:** Xiaohong Lan, Qinyan Wu.

**Formal analysis:** Yang Yang.

**Software:** Xuqing Chen.

**Supervision:** Xuqing Chen, Yuekun Wang.

**Validation:** Yuekun Wang.

**Writing – original draft:** Bo Wu.

**Writing – review & editing:** Bo Wu, Yuekun Wang.
